# Discovery of super soft-drug modulators of sphingosine-1-phosphate receptor 1

**DOI:** 10.1016/j.bmcl.2018.07.044

**Published:** 2018-10-15

**Authors:** Mark Bell, David Foley, Claire Naylor, Colin Robinson, Jennifer Riley, Ola Epemolu, Paul Scullion, Yoko Shishikura, Elad Katz, W.H. Irwin McLean, Paul Wyatt, Kevin D. Read, Andrew Woodland

**Affiliations:** a The Drug Discovery Unit, Biological Chemistry and Drug Discovery, University of Dundee, Dundee DD1 4HN, UK; b Dermatology and Genetic Medicine, Biological Chemistry and Drug Discovery, University of Dundee, Dundee DD1 5EH, UK

**Keywords:** S1PR1, Soft-drug, Plasma stability, Psoriasis, Topical

## Abstract

The oral S1PR1 agonist ponesimod demonstrated substantial efficacy in a phase II clinical trial of psoriasis. Unfortunately, systemic side effects were observed, which included lymphopenia and transient bradycardia. We sought to develop a topical soft-drug S1PR1 agonist with an improved therapeutic index. By modifying ponesimod, we discovered an ester series of S1PR agonists. To increase metabolic instability in plasma we synthesised esters described as specific substrates for paraoxonase and butyrylcholinesterases, esterases present in human plasma.

## Highlights

• Identification of soft drug S1PR agonists.
• Development of a process for the evaluation of topical esterase soft drugs.
• Study skin and plasma esterase SAR.

## Graphical abstract

**Figure:**
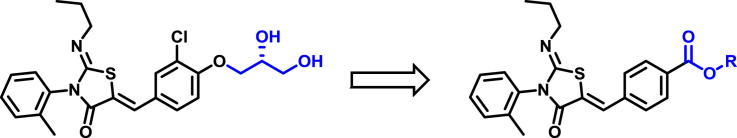

## Introduction

Psoriasis is a common chronic inflammatory skin disease that affects 2% of the population.^1^ 52.3% of patients were dissatisfied with current treatments in a recent survey from the National Psoriasis Foundation in the US.^1^ Recently approved biological drugs targeting disease relevant receptors, such as secukinumab^2^ or ixekizumab^3^ for interleukin (IL)-17 and ustekinumab for IL-12/23^4^ have brought great benefit to patients with severe symptoms of the disease. However, a need remains for safe, convenient, efficacious therapies for mild and moderate psoriasis.

**Fig. 1:**
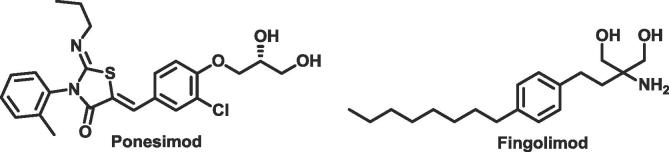
Selected S1PR1 modulators.

Sphingosine-1-phosphate receptor (S1PR) agonists are of interest to the pharmaceutical industry, due to their potential to treat diseases of the immune system such as psoriasis and multiple sclerosis as well as cancer.^5^, ^6^ S1PR agonists, such as fingolimod and ponesimod (Fig. 1), initially activate sphingosine-1-phosphate receptors, but subsequently trigger receptor internalisation. This shuts down the sphingosine 1-phosphate signalling pathway, which then prevents the maturation and migration of lymphocytes.^7^ In 2010 fingolimod was approved for the treatment of relapsing/remitting multiple sclerosis and is the only S1PR1 agonist approved to date.^8^

In a phase 2 study in plaque psoriasis, a 40 mg oral dose of ponesimod, led to a 75% reduction in psoriasis area and severity index (PASI) score in 77% of the patients.^9^ This level of efficacy is competitive with other small molecules in development to treat psoriasis (apremilast^10^, tofacitinib^11^ and sotrastaurin^12^). Ponesimod was not however progressed into phase 3, possibly due to safety concerns. Oral use of pan-S1PR and S1PR1 selective modulators is associated with several severe side effects such as lymphopenia, bradycardia and dyspnoea, which limits their utility as therapies for non-life threatening chronic diseases.^5^

In mice, topical application of the S1PR agonist fingolimod led to a reduction in the number of Langerhans cells migrating to the lymph node.^13^ Work by Griffiths and co-workers has shown that Langerhans cell migration is higher in involved areas of patient’s skin than in non-involved areas of skin.^14^, ^15^ We hypothesised that local inhibition of Langerhans cell migration, through the effects of topical S1PR agonists, could restore a normal skin phenotype to patients with psoriasis.

Due to the potential of S1PR agonists to act as efficacious therapies, for the treatment of psoriasis, we decided to embark on developing a topical S1PR1 modulator using a fast-follower approach, inspired by ponesimod. Topical therapies apply drugs directly to the site of action and can lead to a reduction in the systemic exposure of a drug when compared to oral dosing. However, due to the chronic nature of psoriasis and the need to be able to treat large body surface areas (>10%), there is a danger that topical application could lead to biologically relevant systemic drug concentrations. To maximise the therapeutic index we decided to develop a soft drug.^16^, ^17^ Soft drugs are locally active, in this case in the skin, but then undergo rapid systemic metabolism, to metabolites, which are either inactive or rapidly cleared from the systemic circulation.^18^

Esters can be metabolised in liver by esterases and cytochrome P450 enzymes.^19^ However, most tissues are capable of metabolising an ester. Importantly, human blood contains a wide range of esterases and the rate of ester hydrolysis in blood can lead to ultra-rapid clearance,^20^ what we term a super-soft drug. Because S1PR agonists induce transient bradycardia, and as blood returning to circulation from skin passes through the heart before reaching the liver, we felt that a very rapid blood clearance mechanism could potentially discharge the cardiotoxicity risk. To select compounds which would be likely to demonstrate the desired pharmacokinetic profile, we targeted a human plasma stability half-life of <5 min. We chose to first develop benzoate ester analogues of ponesimod to determine if they could demonstrate potential as super-soft drugs. The desired compound would have S1PR pXC_50_ of >7 and a logP in the range of 2–4 which is believed to be optimal for skin penetration.^21^

**Scheme 1:**
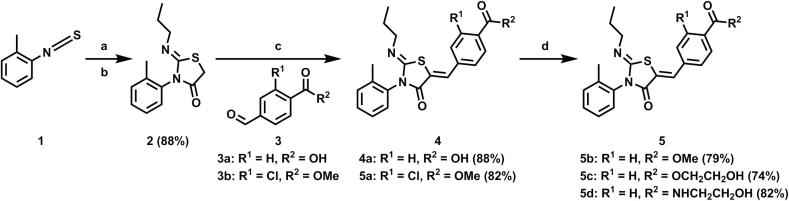
Reagents and conditions for preparation of compounds **4a** and **5a**–**d**: (a) *n*-PrNH_2_ (1 eq), CH_2_Cl_2_, rt (b) 2-bromoacetyl bromide (1 eq) and pyridine (2 eq), CH_2_Cl_2_, 0 °C to rt (two steps) (c) aldehyde (1 eq), NaOAc (2 eq), AcOH, 65 °C (d) alcohol (5 eq), HOAt (1.5 eq), EDC (1.5 eq), DIPEA (2.5 eq), CH_2_Cl_2_, rt. eq: equivalent; rt: room temperature; HOAt: 1-Hydroxy-7-azabenzotriazole; EDC: 1-Ethyl-3-(3-dimethylaminopropyl)carbodiimide; DIPEA: *N*,*N*-Diisopropylethylamine.

Compounds **4a** and **5a**–**d** were synthesised as shown in Scheme 1. The (*Z*)-2-(propylimino)-3-(o-tolyl)thiazolidin-4-one core **2** was synthesised utilising a one-pot, two step reaction. Only the Z isomer was observed. *N*-propylamine was reacted with 1-isothiocyanato-2-methylbenzene **1** to give the resulting thiourea, which was condensed with 2-bromoacetyl bromide followed by addition of pyridine to furnish the desired thiazolidin-4-one **2**. This core was then condensed with the appropriately substituted benzaldehyde **3a**–**b** to furnish **4a** and **5a**. Finally, esterification or amidation of **4a** gave the desired products. Based on the configurational analysis by X-ray, which characterised crystalline analogues of ponesimod as *Z*,*Z*^22^, all structurally related compounds **4a**, **5a**–**d**, **11**, **12a**–**f** and **15a**, **b** were assigned to the *Z*,*Z*-isomer.

We selected human plasma stability as a model for human blood metabolism. Esters **5a**–**c** and amide **5d** demonstrated half-lives of >180 min in human plasma, far greater than the <5 min we were aiming for. S1PR1 activity was measured using a PathHunter β-Arrestin recruitment assay.^23^ All of the ester and amide compounds in Table 1 were active and had pIC_50_ values ranging from 6.5 to 7.0, with the exception of compound **5a** where a chlorine ortho to the ester provided 100-fold improvement in activity compared to **5b**. Gratifyingly, the parent carboxylic acid **4a**, which is the desired metabolite of our esters/amides was less active, giving a pIC_50_ of <6 and meeting our targeted criteria for producing inactive metabolites.

**Table 1:** Effect of R^2^ substitution on S1PR1 activity, kinetic aqueous solubility and human plasma stability.

**Figure:**
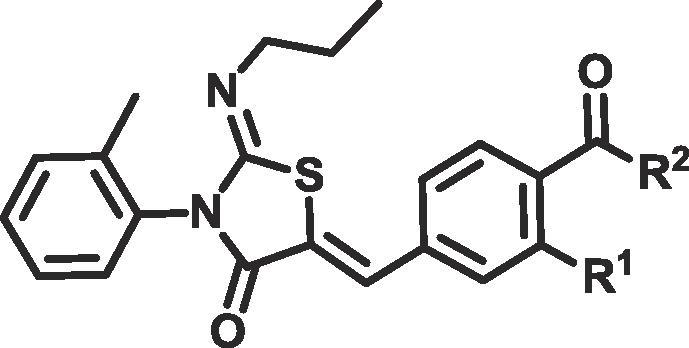

Compound	R^1^	R^2^	Kinetic solubility (µM)^a^	CHI logD^b^	S1PR1 pIC_50_^c^	H Plasma Stability (half-life min)^d^
Ponesimod	–	–	110	3.1	7.9 (0)	>180
**5a**	Cl	OMe	39	>4.4	8.6 (0.1)	>180
**5b**	H	OMe	28	>4.4	6.5 (0)	>180
**4a**	H	OH	163	1.0	<6 (0)	–
**5c**	H	OCH_2_CH_2_OH	110	3.4	6.9 (0)	>180
**5d**	H	NHCH_2_CH_2_OH	>250	2.3	7.0 (0)	>180

a The aqueous solubility of the test compounds was measured using laser nephelometry.

b Reverse-phase HPLC method to determine the chromatographic hydrophobicity index (CHI).

c All pIC_50_s reported in this table correspond to n of 2, reported as their geometric mean. The range of pIC_50_ values is provided in brackets.

d Incubated in human plasma at 37 °C.

Aqueous solubility is an important parameter for topically applied drugs. Increasing aqueous solubility, should directly increase skin penetration rates.^24^ Increasing aqueous solubility also increases the range of formulation options that can be used in pre-clinical development. We were pleased to see that introduction of a hydrogen bond donor at R^2^ in **5c** led to a 4-fold increase in solubility relative to **5b**. Changing the ester linker in **5c** to an amide in **5d** further improved solubility to >250 µM, probably due to improvements in lipophilicity (3.4 vs 2.3 respectively).

**Fig. 2:**
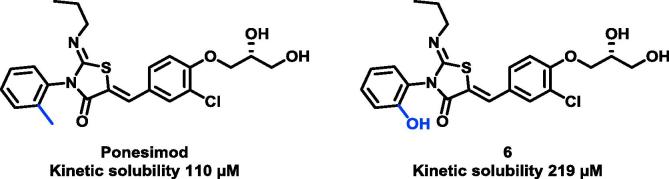
Improvements in aqueous kinetic solubility from introduction of *o*-phenol group.

Encouraged by the solubility and activity data, we focused our efforts on optimising the metabolic profile of the esters. At this time, we identified that switching from the *o*-tolyl group used in ponesimod to an *o*-phenol **6** increased aqueous solubility 2-fold, presumably due to the reduction in lipophilicity (Fig. 2). As **6** was equipotent to ponesimod (pIC50 7.9) and to maximise compound solubility, subsequent compounds incorporated the *o*-phenol group.

**Scheme 2:**
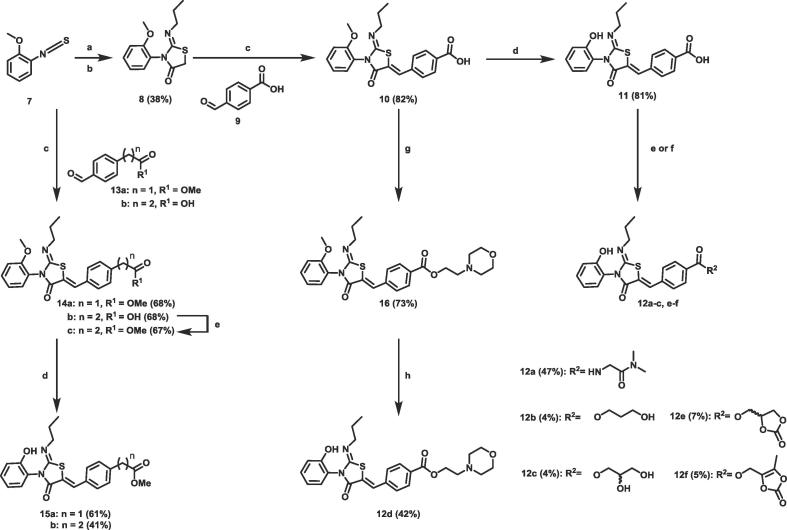
Reagents and conditions for preparation of compounds **11**, **12a**–**f** and **15a**, **b**: (a) *n*-PrNH_2_ (1 eq), CH_2_Cl_2_, rt (b) 2-bromoacetyl bromide (1 eq) and pyridine (2 eq), CH_2_Cl_2_, 0 °C to rt (two steps) (c) aldehyde (1 eq), NaOAc (2 eq), AcOH, 85 °C (d) BBr_3_ (8 eq), CH_2_Cl_2_, −78 °C to rt (e) For **12a**–**c**. amine (3 eq) or alcohol (5 eq), HOAt (1.5 eq), EDC (1.5 eq), DIPEA (2.5 eq), CH_2_Cl_2_, rt (f) For **12d**–**f**. alcohol, alkyl chloride or alkyl tosylate (1.1–1.5 eq) K_2_CO_3_ (1.5 eq), NaI (0.1 eq), DMF, 70 °C (g) NEt_3_ (3 eq), EDC (1.1 eq), HOAt (1.1 eq), alcohol (1.5 eq) rt (h) BBr_3_ (5 eq), CH_2_Cl_2_, −78 °C to rt. eq: equivalent; rt: room temperature; HOAt: 1-Hydroxy-7-azabenzotriazole; EDC: 1-Ethyl-3-(3-dimethylaminopropyl)carbodiimide; DIPEA: *N*,*N*-Diisopropylethylamine; DMF: dimethyl formamide.

Compounds **11**, **12a**–**c**, **15a** and **15b** were synthesised using the route shown in Scheme 2. **12a**–**c** were more soluble than ponesimod, probably due to significant improvements in CHI logD (Table 2). As expected a clear trend can be seen between low lipophilicity and improved solubility. Homologation of the ester group (**15a**, **b**) showed increased lipophilicity (3.2 and 3.5 respectively) and a commensurate reduction in solubility. However, as with **5a**–**d**, amide **12a** and esters **12b**–**c** also showed a lack of metabolism in our human plasma stability assay (Table 2).

**Table 2:** Effect of R^1^ substitution on kinetic aqueous solubility and human plasma stability.

**Figure:**
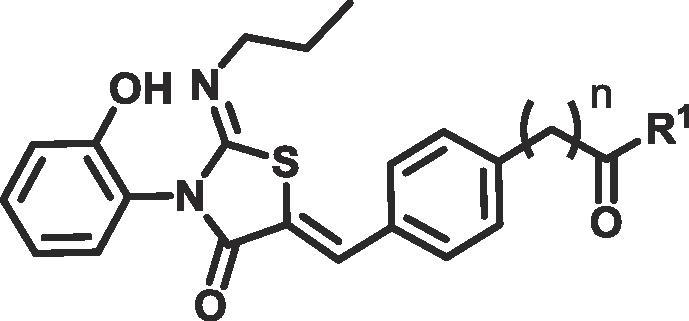

Compound	n	R^1^	Kinetic solubility (µM)^a^	CHI logD^b^	H Plasma Stability (half-life min)^c^
Ponesimod	–	–	110	3.1	>180
**12a**	0	NHCH_2_CONMe_2_	219	1.9	>180
**12b**	0	OCH_2_CH_2_CH_2_OH	183	2.3	>180
**12c**	0	OCH_2_CH(±CH_2_OH)OH	>250	1.8	>180
**15a**	1	OMe	155	3.2	>180
**15b**	2	OMe	79	3.5	>180

a The aqueous solubility of the test compounds was measured using laser nephelometry.

b Reverse-phase HPLC method to determine the chromatographic hydrophobicity index (CHI).

c Incubated in human plasma at 37 °C.

As simple esters had shown themselves to be stable to human plasma, we turned our attention to ester functional groups which had been shown to be unstable in human plasma in the literature (half-life < 5min).^20^ The most successful studies focused on optimising metabolism by the esterases paraoxonase^25^ and butyrylcholinesterases^26^, which are mainly expressed in blood plasma.

**Fig. 3:**
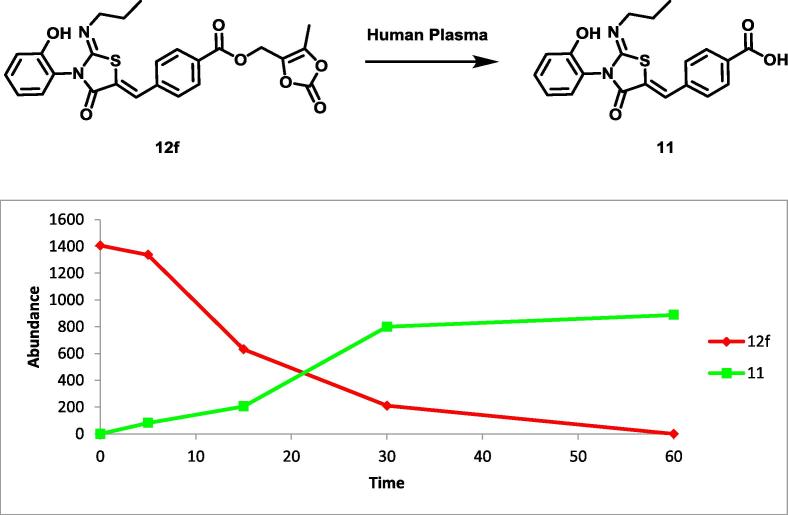
Profile of compound **12f** being hydrolysed to **11** in human plasma. **12f** was incubated in human plasma at 37 °C. The experiment was conducted as an n of one to provide a qualitative analysis.

We synthesised compounds **11** and **12d**–**f** using the route shown in Scheme 2, which all contain ester groups that are unstable in human plasma when attached to other scaffolds. Although **12d** or **12e** did not show sufficient clearance in human plasma we were pleased to identify compound **12f** containing a (2-oxo-1,3-dioxolan-4-yl)methyl ester which is rapidly degraded in human plasma (Table 3). The half-life of this ester in human plasma was 9 min and metabolite identification confirmed the conversion to the inactive parent acid **11** (Fig. 3).

**Table 3:** Effect of R^1^ substitution on kinetic aqueous solubility and human plasma stability.

**Figure:**
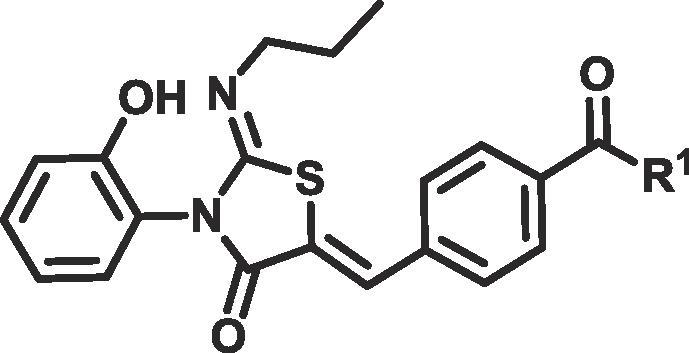

Compound	R^1^	H Plasma Stability (half-life min)^a^	Kinetic solubility (µM)^b^	CHI logD^c^	H Skin S9 (half-life min)^d^	S1PR1 pIC_50_^e^
**11**	OH	>180	–	–	–	<6 (0)
**12d**		180	117	3.0	152	–
**12e**		61	79	2.8	40	–
**12f**		9	79	3.3	23	<6 (0)

a Incubated in human plasma at 37 °C.

b The aqueous solubility of the test compounds was measured using laser nephelometry.

c Reverse-phase HPLC method to determine the chromatographic hydrophobicity index (CHI).

d Stability measured in skin S9 over 180 mins in the presence of enzymatic cofactors.

e All pIC_50_s reported in this table correspond to n of 2, reported as their geometric mean. The range of pIC_50_ values is provided in brackets.

Unfortunately **12f** was also rapidly metabolised in human skin S9 fraction with a half-life of just 23 min, far faster than the desirable half-life of >6 h that we target for once daily dosing. In fact, there are suggestions of a correlation between the relative stability of **12d**–**f** in human plasma and skin S9 fraction. It may be that the esterases responsible for the metabolism of **12f** are present in both human plasma and skin S9 fraction or that skin and plasma esterases have similar substrate affinities. Unfortunately, **12f** was also inactive as a S1PR1 modulator (Table 3).

In summary, using a fast follower approach, we identified a series of ester S1PR1 modulators. Further optimisation led to improved solubility and identification of ester **12d** that is plasma unstable. Unfortunately, the ester is inactive and is metabolised in human skin models. Further efforts will focus on identifying an active, skin stable compound that is rapidly metabolised systemically will be disclosed in due course.
